# Hyperendemic carbapenem-resistant *Acinetobacter baumannii* at a hospital in Botswana: Insights from whole-genome sequencing

**DOI:** 10.1017/ash.2023.395

**Published:** 2023-09-29

**Authors:** Jonathan Strysko, Tefelo Thela, Janet Thubuka, Tichaona Machiya, Jack Mkubwa, Celda Tiroyakgosi, Moses Vurayai, Kgomotso Kgomanyane, Tlhalefo Dudu Ntereke, Tshiamo Zankere, Kwana Lechiile, Teresia Gatonye, Chimwemwe Tembo, Naledi Betsi Mannathoko, Margaret Mokomane, Andries Feder, Melissa Richard-Greenblatt, David Goldfarb, Carolyn McGann, Susan Coffin, Ebbing Lautenbach, Corrado Cancedda, Dineo Bogoshi, Anthony Smith, Paul Planet

## Abstract

**Background:** Carbapenem-resistant *Acinetobacter baumannii* (CRAB) has emerged as a major cause of bloodstream infection among hospitalized patients in low- and middle-income countries (LMICs). CRAB infections can be difficult to treat and are devastating in neonates (~30% mortality). CRAB outbreaks are hypothesized to arise from reservoirs in the hospital environment, but outbreak investigations in LMICs seldom incorporate whole-genome sequencing (WGS). **Methods:** WGS (Illumina NextSeq) was performed at the National Institute for Communicable Diseases (South Africa) on 43 preserved *A. baumannii* isolates from a 530-bed referral hospital in Gaborone, Botswana, from March 2021–August 2022. This included 23 blood-culture isolates from 21 unique patients (aged 2 days–69 years) and 20 environmental isolates collected at the 36-bed neonatal unit in April–June 2021. Infections were considered healthcare-associated if the culture was obtained >72 hours after hospital arrival (or sooner in inborn infants). Blood cultures were incubated using an automated system (BACT/ALERT, BioMérieux) and were identified using manual methods. Environmental isolates were identified using selective or differential chromogenic media (CHROMagarTM). Taxonomic assignment, multilocus sequence typing (MLST), antimicrobial resistance gene identification, and phylogenetic analyses were performed using publicly accessible analysis pipelines. Single-nucleotide polymorphism (SNP) matrices were used to assess clonal lineage. **Results:** All 23 blood isolates and 5 (25%) of 20 environmental isolates were confirmed as *A. baumannii*; thus, 28 *A. baumannii* isolates were included in the phylogenetic analysis. MLST revealed that 22 (79%) of 28 isolates were sequence type 1 (ST1), including all 19 healthcare-associated blood isolates and 3 (60%) of 5 environmental isolates. Genes encoding for carbapenemases (*bla*NDM-1, *bla*OXA-23) and biocide resistance (*qac*E) were present in all 22 ST1 isolates; colistin resistance genes were not identified. Phylogenetic analysis of the ST1 clade demonstrated spatial clustering by hospital unit. Related isolates spanned wide ranges in time (>1 year), suggesting ongoing transmission from environmental sources (Fig. 1). An exclusively neonatal clade (0–2 SNPs) containing all 8 neonatal blood isolates was closely associated with 3 environmental isolates from the neonatal unit: a sink drain, bed rail, and a healthcare worker’s hand. **Conclusions:** WGS analysis of clinical and environmental *A. baumannii* revealed the presence of unit-specific CRAB clones, with evidence of ongoing transmission likely driven by persistent environmental reservoirs. This research highlights the potential of WGS to detect hospital outbreaks and reaffirms the importance of environmental sampling to identify and remediate reservoirs (eg, sinks) and vehicles (eg, hands and equipment) within the healthcare environment.

**Figure:**
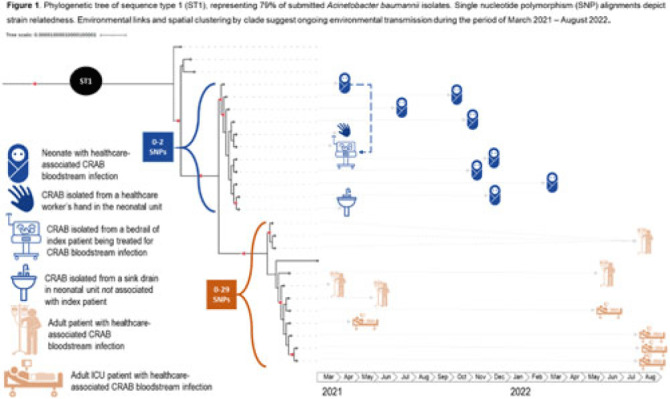

**Disclosures:** None

